# Current situation and influencing factors of each turnover of kindergarten teachers – a questionnaire survey

**DOI:** 10.3389/fpsyg.2024.1321441

**Published:** 2024-02-12

**Authors:** Xiaoling Ren, Zhonglian Yan, Zedong Zhang, Jiewen Chen, Yun Tian

**Affiliations:** ^1^School of Education, Beihua University, Jilin, China; ^2^Faculty of Education, Northeast Normal University, Changchun, China

**Keywords:** teacher turnover, kindergarten teacher, influencing factors, current situation, questionnaire survey

## Abstract

**Objective:**

Frequent teacher turnover may damage the development of teachers and the regular operation of kindergartens. This original research presented kindergarten teachers’ first, second, and third turnover rates and occurrence times. This research analyzed the relationship between socio-demographic variables and the varying frequency of kindergarten teacher turnover. These data were used to investigate the characteristics of first, second, and third kindergarten turnover. This research evaluated kindergarten teachers’ occupational ambition, emotional attachment, and self-efficacy. Likewise, this research also analyzed the social context, organizational support, management mechanism, reward, and occupational stress of kindergarten. These data were used to determine the key factors affecting kindergarten teachers’ turnover.

**Methods:**

This research recruited 1,118 kindergarten teachers (mean age = 31.67, sd = 5.02; 3.85% male, 96.14% female) from China. Based on the existing scales, this research developed the Questionnaire of Kindergarten Teachers’ Turnover and Influencing Factors for the survey. Kindergarten teachers reported basic information and the impact factors of their first, second, and third turnover through online questionnaires. The Chi-square test was used to analyze the correlation between socio-demographic variables and different frequencies of kindergarten teacher turnover. The binary logistic regression explored the eight factors affecting kindergarten teachers’ first, second, and third turnover.

**Results:**

The results showed that 43.65% of kindergarten teachers had resigned. In detail, 25.60% of kindergarten teachers resigned once, 10.64% of kindergarten teachers resigned twice, and 8.41% of kindergarten teachers resigned thrice. Gender and marital status were significantly correlated with the three frequencies of kindergarten teacher turnover. Occupational stress, reward, management mechanisms, and ambition consistently affected kindergarten teachers’ first, second, and third turnover.

**Conclusion:**

The relevant management departments should pay attention to the high turnover rate of kindergarten teachers and put forward more strategies to improve their stability. Women and married can be favored in the recruitment of kindergarten teachers. It is crucial to reduce pressure and improve rewards for kindergarten teachers. Also, kindergartens should provide the space to display teachers’ talents and improve management mechanisms. These results provide empirical support for proposing effective policies to promote the stability of kindergarten teachers’ construction.

## 1 Introduction

Since 2010, the “Ministry of Education of the People’s Republic of China” has issued policies to promote the popularization of kindergartens and expand the kindergarten teacher workforce. The kindergarten enrolment rate increased by 26.2% during the 12 years (Hu and Cai, 2022). Chinese government suggested that the ratio between kindergarten teachers and children should be at least 1:9. Still, this ratio has reached 1:17 in 2021 (Guan, 2021). Therefore, kindergarten teachers has a high turnover rate. For example, from 2013 to 2018, there was a 12–14% kindergarten teacher turnover rate in Shenzhen, China (Hai et al., 2020). The kindergarten teachers in Hubei province had a 29.7% turnover rate (Gong et al., 2020). Teachers’ first jobs in Chile lasted only 3.1 years, and 20% of teachers left the teaching profession during their 5-year internship (Palma-Vasquez et al., 2022). Approximately 50% of teachers leave within the first five years of employment in America, England, and Australia (Ewing and Manuel, 2005; Borman and Dowling, 2008). Accordingly, increasing the kindergarten teachers’ stability is a key to solving the vacancies for kindergarten teachers (Griffin et al., 2014). Teachers’ stability is affected by teacher development, kindergarten administrative processes, and organizational culture (Zoglu, 2015). Solving the instability of teachers’ careers is crucial for children’s development and kindergarten teachers’ vacancies (Kim and Park, 2018). Therefore, analyzing the impact factors of kindergarten teacher turnover is necessary. However, previous studies focused on the situation and reasons for single or integrated turnover (Rosanne et al., 2020). The situation and impact factors of different frequency turnover remain unclear. This research explored the turnover rate and times of kindergarten teachers in China. We analyzed the characteristics of kindergarten teachers who may continue to resign. Finally, we discussed the factors that lead to frequent teacher turnover. This study enriched the research on kindergarten teacher turnover and provided empirical support to improve the stability of kindergarten teachers.

### 1.1 The conception of teacher turnover

The teacher turnover was divided into leavers, stayers, and movers (Vagi and Pivovarova, 2020). Leavers refer to teachers who leave the profession and no longer engage in educational work. Stayers are teachers who leave their current teaching profession and move to another teaching profession in their original school. Movers are teachers who leave their current school to work in another school but maintain their teaching profession. Compared with stayers, movers are more unfavorable to the teacher workforce construction (Borman and Dowling, 2008). Many teachers leave their schools before their performance reaches peak (Darling-Hammond, 2003). As a result, teachers in schools serving the most disadvantaged students may never achieve the expected level of professionalism. This phenomenon affects the teaching quality of schools and widens the inequality gap in different schools (Cabezas et al., 2017). Moreover, previous studies divided teacher turnover into individual and guided turnover (Van Geffen and Poell, 2014). Individual turnover is the active behavior of teachers after weighing advantages and disadvantages. Guided turnover refers to the state’s measures for teachers to move between schools or dismiss teachers. Teachers’ stability and orderly turnover could be more conducive to education health and sustainable development (Avalos and Valenzuela, 2016). Furthermore, some studies divide teacher turnover indirectly and directly (Palma-Vasquez et al., 2022). Indirect turnover refers to the possible rotation or abandonment of teachers in the future, which indicates the teachers’ turnover intentions. Direct turnover refers to the actual act of teacher rotation or abandonment. This research focuses on movers, individuals, and direct turnover. Kindergarten teacher turnover in our study refers to the active turnover behavior of kindergarten teachers from the current school to another kindergarten. This turnover does not include the passive turnover of kindergarten teachers (such as being fired, administrative guidance turnover, and teacher turnover caused by the government).

### 1.2 The current situation of kindergarten teacher turnover

Some researchers measured kindergarten teacher turnover in many countries. For example, a study tracked Louisiana, America’s 40% teacher turnover rate (Doromal et al., 2022). Only 30.7% of the 1,137 kindergarten teachers in Nagasaki Prefecture, Japan, were willing to work in kindergartens for over five years (Tayama et al., 2019). About 61.4% of kindergarten teachers in the United States would leave their jobs when the classroom resources were insufficient to meet the job’s demands (Lambert et al., 2019). In 2020, the turnover rate of private kindergarten teachers in China has been reached 41% (Yang et al., 2021). In addition, several studies analyzed the correlation between socio-demographic variables and teacher turnover, including gender, educational background, and school type. For example, gender was significantly related to teachers’ turnover intention (Van Droogenbroeck and Spruyt, 2015). A study found that male teachers were likelier to leave their profession than female teachers (Quartz et al., 2005). Moreover, there was a significant correlation between school type and teacher turnover, such as the high turnover rate in private schools (Hall et al., 2013). However, a study found similar turnover rates in public and private schools (Cassettari et al., 2014). This phenomenon may be attributed to the different samples and measurement methods. Furthermore, a study proposed that educational background was positively related to teachers’ turnover intention (An and Cao, 2017). Teaching experience was positively correlated with the teaching satisfaction of kindergarten teachers, thus reducing the turnover of kindergarten teachers (Yin et al., 2023). However, most of these studies focus on kindergarten teachers’ turnover intentions, not turnover behavior.

### 1.3 Influencing factors of kindergarten teacher turnover

Previous studies suggest that teacher turnover is affected by multiple factors. The factors affecting teacher turnover can be divided into personal, working conditions, and organizational structure characteristics. Besides socio-demographic variables, personal characteristics include emotional attachment, teaching skills, job satisfaction, professional commitment, self-efficacy, and occupational ambition. Generally, teachers prefer to work near their families, and teacher turnover might increase if their families are far from their workplace (Reininger, 2012). Moreover, the experienced teachers are more likely to resign (Goldhaber et al., 2016). Teachers’ job satisfaction significantly predicted teacher retention (Tickle et al., 2011). A study found that identity perception significantly predicted teacher turnover (Horvath et al., 2018). Professional commitment could reduce teacher turnover (Tiplic et al., 2015). A study showed that self-efficacy was crucial for teacher turnover (Swanson, 2014). Professional development opportunities increased teacher turnover, and teachers left their jobs to pursue better careers and lives (Shin et al., 2006; Gutman, 2019).

Working condition characteristics include school social context, reward, and occupational stress. Excessive occupational stress can pressure teachers and lead to turnover (Deangelis and Presley, 2011). The high work pressure can lead to burnout of kindergarten teachers, which would inevitably accelerate the kindergarten teacher turnover (Li et al., 2020; Chen et al., 2023). Rewards negatively predicted teacher turnover (Berlinski and Ramos, 2020). Nevertheless, some studies found that relative salary was an essential determinant of teacher turnover (Bradley et al., 2012). A study showed that teachers might leave due to the poor social context of school (Barbieri et al., 2011). Likewise, the economic and geographical conditions of the school area were the main factors affecting teacher turnover (Zoglu, 2015).

Organizational structure characteristics include organizational support and management mechanisms. A study proposed that organizational support was essential to prevent teacher turnover (Tiplic et al., 2015). A study found a positive correlation between school management and teacher retention (Boe et al., 2008). Moreover, accountability, performance evaluation, and incentives can predict teacher turnover (Adnot et al., 2017; Ryan et al., 2017). Teachers’ happiness in school predicted student-related, work-related, and personal burnout, which might influence teacher turnover (de Stasio et al., 2017). However, these empirical studies mainly analyzed the effect of a single aspect on teacher turnover. The impact factors in each turnover frequency of kindergarten teachers need to be further explored.

### 1.4 Measurement of influencing factors of teacher turnover

The quantitative approach is widely used to measure the impact factors of teacher turnover (Palma-Vasquez et al., 2022). There are two ways to evaluate teacher turnover and its impact factors. One of these methods focuses on teachers’ turnover intentions and their impact factors. These studies used scales to measure the same or similar aspects and mainly measured the 1–3 impact factors of teacher turnover (Kukla-Acevedo, 2009; Renzulli et al., 2011). Another method focuses on teachers’ turnover behavior and its impact factors. These studies used information from national statistical items or self-developed scales (Yesil, 2012; Buckman, 2021). The self-developed scales were mostly used to measure teachers’ single turnover impact factors. In this study, we used multiple scales to evaluate the effects of various factors on kindergarten teacher turnover. However, measuring the impact factors with multiple scales will consume much time and energy. Therefore, the effectiveness of the measurement method needs to be considered when measuring the impact factors of each turnover of kindergarten teachers.

### 1.5 Present research

This research screened eight factors influencing kindergarten teacher turnover: occupational ambition, emotional attachment, self-efficacy, social context of kindergarten, organizational support, management mechanism, reward, and occupational stress. These factors were selected by literature analysis and pre-survey. This research explores the influence of these eight factors on each turnover frequency. Occupational ambition is the ideal and goal of kindergarten teachers in occupational development (Resnick, 2017). Emotional attachment is the tendency of kindergarten teachers to seek and maintain an attachment object, which can provide a stable sense of physical and mental security (Ryan et al., 2017). Self-efficacy refers to kindergarten teachers’ perception and belief to organize and implement relevant educational activities (Ladd, 2011). The social context of kindergarten is the credibility, influence, and appeal established by kindergarten (Hancock and Scherff, 2010). Organizational support is the kindergarten’s recognition of teachers’ contributions and benefits (Fulbeck, 2014). Management mechanism refers to the rules and regulations to ensure the regular operation of kindergartens (Barbieri et al., 2011). Rewards refer to the salary and benefits of kindergarten teachers (Hancock and Scherff, 2010). Occupational stress refers to the negative emotions of kindergarten teachers. This stress includes stress from outside, themselves, and their working environment (An and Cao, 2017).

This research tried to present kindergarten teachers’ first, second, and third turnover rates and times. Moreover, this research explored the relationship among genders, educational background, kindergarten type, marital status, and kindergarten teachers’ first, second, and third turnover. Based on previous studies, this research hypothesizes:

H1. Genders, marital status, type of kindergarten, and educational background are significantly correlated with each turnover of kindergarten teachers.

Furthermore, this research obtained the influential factors of kindergarten teacher mobility, including occupational ambition, emotional attachment, self-efficacy, social context of kindergarten, organizational support, management mechanism, reward, and occupational stress. This research tried to discuss the factors affecting the continuous turnover of kindergarten teachers. This research hypothesizes:

H2. Occupational ambition, emotional attachment, self-efficacy, the social context of kindergarten, organizational support, management mechanism, reward, and occupational stress significantly affect each turnover of kindergarten teachers.

## 2 Materials and methods

### 2.1 Participants

This research conducted online questionnaires among kindergarten teachers in Jilin, Liaoning, Heilongjiang, Beijing, Shandong, and Jiangxi Provinces in China. Kindergarten teachers reported the variables through online questionnaires. One thousand two hundred ninety-four questionnaires were distributed through simple random sampling, and 1,118 valid questionnaires were recovered, with an effective rate of 86.40%. Among them were 185 kindergarten teachers from Jilin Province, 181 from Liaoning Province, 143 from Heilongjiang Province, 214 from Beijing, 218 from Shandong Province, and 177 from Jiangxi Province. The age of non-turnover kindergarten teachers was 27.68 ± 5.31 (mean ± sd, *n* = 630), and their teaching experience was 4.63 ± 4.18 (mean ± sd, *n* = 630) years. The age of kindergarten teachers’ first turnover was 26.95 ± 4.59 (mean ± sd, *n* = 488), and their teaching experience was 2.03 ± 1.04 (mean ± sd, n = 488) years. The age of kindergarten teachers’ second turnover was 30.04 ± 4.12 (mean ± sd, *n* = 213), and their teaching experience was 4.71 ± 1.79 (mean ± sd, *n* = 213) years. The age of kindergarten teachers’ third turnover was 34.00 ± 5.09 (mean ± sd, *n* = 94), and their teaching experience was 8.74 ± 1.87 (mean ± sd, *n* = 94) years. Table 1 shows the basic information on kindergarten teachers’ turnover at different frequencies.

**Table 1:** The basic information of kindergarten teachers’ turnover at different frequencies.

	Non-turnover	First turnover	Second turnover	Third turnover
Female	620	455	197	83
Male	10	33	16	11
Married	242	160	110	62
Unmarried	388	328	103	32
Private kindergartens	378	162	91	54
Public kindergartens	252	326	122	40
Bachelor’s degree or above	316	252	104	44
Three-year college degree or below	314	236	109	50
Urban kindergarten	426	336	161	63
Rural kindergarten	204	152	52	31

### 2.2 Measures

Based on the existing scales, This research developed a Questionnaire of Kindergarten Teachers’ Turnover and Influencing Factors for the survey. This questionnaire was divided into primary information and information on influencing factors. The basic information section consists of 12 fill-in-the-blank questions. It mainly obtains information on kindergarten teachers’ first, second, and third turnover, such as teaching age, educational background, marital status, and kindergarten type. The influencing factors information section consisted of 24 items. These items come from scales compiled by the researchers. However, considering the time and energy of kindergarten teachers to fill in the questionnaire, this research only selected some items from scales compiled by the researchers.

Chan et al. (2012) compiled the Occupational Ambition Scale (Chan et al., 2012), which was translated into Chinese and validated by Li (2015) and adapted to measure the occupational ambition of employees in different occupations (Li, 2015). This research selected three items from this scale to measure the occupational ambition of kindergarten teachers. The Cronbach’s alpha of this subscale was 0.84. The subscale reported a good Cronbach’s alpha.

Brennan et al. (1998) compiled the Experience in Close Relationships Inventory (Brennan et al., 1998), which was translated, revised, and validated by Li (2006) to be suitable for assessing the emotional attachment of Chinese adults (Li, 2006). This research selected three items from this scale to measure the emotional attachment of kindergarten teachers. The Cronbach’s alpha of this subscale was 0.81. The subscale reported a good Cronbach’s alpha.

Lian (2007) developed a scale of Chinese teachers’ self-efficacy, which has high reliability (Lian, 2007). This research selected three items from this scale to measure the self-efficacy of kindergarten teachers. The Cronbach’s alpha of this subscale was 0.75. The subscale reported an acceptable Cronbach’s alpha.

Liu (2016) developed the Chinese School Social Context Scale, which has high reliability and validity (Liu, 2016). This research selected three items from this scale to measure the social context of kindergarten teachers. The Cronbach’s alpha of this subscale was 0.84. The subscale reported a good Cronbach’s alpha.

Alatambagan (2014) developed a high-reliability and validity Occupational Stress Scale for kindergarten teachers in China (Alatambagan, 2014). This research selected three items from this scale to measure the occupational stress of kindergarten teachers. The Cronbach’s alpha of this subscale was 0.86. The subscale reported a good Cronbach’s alpha.

Lin et al. (2006) developed the Perceived Organizational Support Scale, which can effectively evaluate the perceived organizational support of Chinese kindergarten teachers (Lin et al., 2006). This research selected three items from this scale to measure the organizational support of kindergarten teachers. The Cronbach’s alpha of this subscale was 0.81. The subscale reported a good Cronbach’s alpha.

You (2021) developed a School Management Mechanism Scale with high reliability and validity for Chinese teachers (You, 2021). This research selected three items from this scale to measure the management mechanism of kindergarten teachers. The Cronbach’s alpha of this subscale was 0.80. The subscale reported a good Cronbach’s alpha.

Wu (2022) developed a highly reliable scale of factors affecting teacher turnover in China, and the sub-scale can be used to evaluate teacher rewards (Wu, 2022). This research selected three items from this scale to measure the reward of kindergarten teachers. The Cronbach’s alpha of this subscale was 0.77. The subscale reported an acceptable Cronbach’s alpha.

Table 2 shows the results of the exploratory factor analysis. Table 3 shows the results of the confirmatory factor analysis. All items were rated on a 5-point Likert scale (1 = ‘very inconsistent’ to 5 = ‘very consistent’)—the fewer scores, the lower the influence on kindergarten teacher turnover. The Cronbach’s alpha of the total sample was 0.77. The scale reported an acceptable Cronbach’s alpha.

**Table 2:** Exploratory factor analysis of the scale of influencing factors of kindergarten teacher turnover.

	Component matrix							
	OS	SC	OA	EA	OR	MM	RE	SE
Q1: I was troubled to relax at work.	0.63							
Q2: My work was very granular and complex.	0.85							
Q3: I encountered many problems in the process of taking care of children.	0.69							
Q4: The kindergarten was incredibly famous in the local area.		0.72						
Q5: The children in the kindergarten were well-developed, and the teachers were easy to carry out their work.		0.69						
Q6: Parents rushed to send their children to our kindergarten for learning and development.		0.83						
Q7: I wanted to reach a high level in my professional field and have much experience.			0.84					
Q8: I sought a high degree of professionalism.			0.54					
Q9: I felt privileged to excel in my field.			0.53					
Q10: It made me feel good to be able to count on someone.				0.72				
Q11: I found that no one could help me when I needed help.				0.82				
Q12: I worried about whether people liked me.				0.67				
Q13: The kindergarten provided me with good working conditions.					0.82			
Q14: When I encountered difficulties in work and life, the kindergarten would help me.					0.77			
Q15: The kindergarten made decisions with my best interests in mind.					0.72			
Q16: The management system of kindergartens was more scientific.						0.81		
Q17: All bureaucratic systems in kindergartens have been reduced to a minimum.						0.68		
Q18: The work assignment in the kindergarten was obvious and reasonable.						0.56		
Q19: The kindergarten paid its teachers a very reasonable salary.							0.82	
Q20: The kindergarten’s salary could give me a good quality of life.							0.55	
Q21: The kindergarten gave me a lot of benefits.							0.67	
Q22: I had less influence on children than their parents.								0.82
Q23: As long as I worked hard, I could change most children with learning difficulties.								0.64
Q24: I could handle work well.								0.45
Eigenvalue	3.84	3.59	3.09	2.32	2.22	1.82	1.45	1.34
% of variance	15.38	14.38	12.36	9.27	8.89	7.27	5.79	5.34

h2, Communality Coefficient; Rotation method, Promax with Kaiser normalization; OS, occupational stress; SC, Social Context of Kindergarten; OA, occupational ambition; EA, Emotional Attachment; OR, Organizational Support; MM, Management Mechanism; RE, Reward; SE, Self-efficacy. Extraction method: Principal axis factoring.

**Table 3:** Confirmatory factor analysis of the scale of influencing factors of kindergarten teacher turnover.

Model fit	
GFI	0.85
RMSEA	0.04
RMR	0.02
CFI	0.93
NFI	0.92

GFI, goodness of fit index; RMSEA, Root Mean Square Error of Approximation; RMR, Root Mean Square Residual; CFI, comparative fit index; NFI, normal of fit index.

### 2.3 Procedure

First, this research developed measurement tools. This research considered that kindergarten teachers need more energy and time to fill out questionnaires. This research wanted to capture the factors affecting kindergarten teacher turnover. This research sorted out 16 factors influencing teacher turnover from the relevant literature. This research designed multiple-choice questions to obtain the factors affecting kindergarten teacher turnover. Meanwhile, This research also designed fill-in-the-blank questions to gain kindergarten teachers’ turnover experience. This research asked 72 kindergarten teachers who had experienced turnover to determine what they considered important factors affecting their turnover. After collating the data, This research identified the eight factors they cited most often. This research found that only a few kindergarten teachers left their jobs more than three times. Therefore, this research analyzed information about kindergarten teachers’ first, second, and third turnovers. Then, This research designed an initial questionnaire based on the relevant scales to obtain basic information and influencing factors of kindergarten teacher turnover. This research distributed the initial questionnaire to 208 kindergarten teachers in Jilin and Heilongjiang Provinces in China. This research conducted an exploratory and confirmatory factor analysis of the data obtained in this survey. According to the analysis results, This research deleted some unsuitable items and retained three suitable items for each key influencing factor, thus forming the final questionnaire. This research recruited kindergarten teachers who participated in these surveys on a voluntary and protective basis.

Second, This research uses the final questionnaire to conduct a formal survey. Before participating in the survey, kindergarten teachers received a letter that provided information on the objectives and procedures of the study. The researchers distributed questionnaires after the kindergarten teachers agreed to participate in this survey. Meanwhile, This research will explain the methods and procedures for filling out the questionnaire. Kindergarten teachers received four questionnaires at the same time, namely, the Questionnaire for Kindergarten Teachers Who Have Not Resigned, the Questionnaire for Kindergarten Teachers Who Resigned Once, the Questionnaire for Kindergarten Teachers Who Resigned Twice, and the Questionnaire for Kindergarten Teachers Who Resigned Thrice. The four questionnaires were similar in content. This research asked kindergarten teachers to recall their situation at each turnover and fill out the corresponding questionnaire. Kindergarten teachers who have never resigned would fill out the first questionnaire to obtain basic information and their perception of eight key influencing factors. Kindergarten teachers with resignation experience might fill out the second to fourth questionnaires. If kindergarten teachers have resigned once, they only need to complete the second questionnaire. If kindergarten teachers have resigned twice, they must complete the second and third questionnaires. If kindergarten teachers have resigned thrice, they must fill in the second, third, and fourth questionnaires.

Third, This research processed the collected data and filtered out invalid data. This research complies with the Declaration of Helsinki. This research would strictly protect the privacy of kindergarten teachers participating in the survey, and the questionnaire would not collect sensitive information such as the names of kindergarten teachers and kindergartens. Moreover, This research ensured that the information obtained was used for statistical analysis. This research confirmed that the information obtained would be disseminated with the consent of the kindergarten teachers. The research was approved by the Ethics Committee of the School of Education, Beihua University (Approval Number: BHSE-2022-313). All participants provided informed consent.

### 2.4 Analytical strategy

Event history analysis is a statistical method to study the occurrence of a series of events and their occurrence time. It investigates the occurrence of events and related influencing factors (Janet and Jones, 2004). These events are varied, such as the termination of a job, the end of a marriage, the arrest of a crime, the end of life, etc. (Janet and Jones, 2004). Researchers can obtain relevant information by asking individuals to recall the moment the event occurred. Therefore, This research used event history analysis to determine kindergarten teachers’ first, second, and third turnover. This research randomly placed three items throughout the questionnaire to reduce social desirability. Individuals who answered two or more of these three items in a contradictory fashion were excluded from the sample. Moreover, This research used G*Power 3.1 software to perform post-hoc power analysis according to the criteria set by Cohen (1973). The power of the Chi-square test analysis ranged from 0.99 to 1. The power of binary logistic regression ranged from 0.87 to 0.96. They were all above the minimum standard of 0.8. This research used SPSS 25.0 software for descriptive statistics, the Chi-square test, and the binary logistic regression. These three statistical approaches were used to present the current situation and influencing factors of kindergarten teachers’ first, second, and third turnover. The dependent variables of the three binary logistic regression were kindergarten teachers in the first, second, and third turnover and non-turnover. The independent variables of the three binary logistic regression were occupational ambition, emotional attachment, self-efficacy, social context of kindergarten, organizational support, management mechanism, reward, and occupational stress kindergarten teachers in the first, second, and third turnover and non-turnover. Furthermore, covariates of the three binary logistic regressions were the genders, educational background, marital status, and kindergarten type of kindergarten teachers in the first, second, and third turnover and non-turnover. The odds ratios (OR) and the 95% confidence intervals were generated. Statistical significance for all analyses was determined at *p* < 0.05.

## 3 Results

### 3.1 The current situation of kindergarten teachers’ different frequency turnover

The number of kindergarten teachers that had no turnover is 56.35%. There, 24.60% of kindergarten teachers have once resigned behavior. The first turnover of kindergarten teachers occurred after 2.03 years of teaching experience. Table 4 shows the Chi-square test results of the different frequency turnover of kindergarten teachers associated with socio-demographic factors. The association between the kindergarten teachers’ first turnover and genders was significant (*p* < 0.05). The association between the kindergarten teachers’ first turnover and marital status was significant (*p* ≤ 0.05). The association between the kindergarten teachers’ first turnover and kindergarten type was significant (*p* < 0.05). The association between the kindergarten teachers’ first turnover and educational background was insignificant (*p* > 0.05).

**Table 4:** The different frequency turnover of kindergarten teachers association with socio-demographic factors.

	First turnover			Second turnover			Third turnover		
	*χ* ^2^	p	Phi	*χ* ^2^	p	Phi	*χ* ^2^	p	Phi
Genders	19.91	0.00	0.13	18.69	0.00	0.15	29.71	0.00	0.20
Marital status	3.78	0.05	0.06	11.45	0.00	0.12	25.48	0.00	0.19
Kindergarten types	79.11	0.00	0.27	19.25	0.00	0.15	0.22	0.64	0.02
Educational background	0.24	0.62	0.02	146.12	0.00	0.44	27.48	0.00	0.23

There were 10.64% of kindergarten teachers who have twice resigned behavior. The second turnover of kindergarten teachers occurred after 4.71 years of teaching experience. As shown in Table 4, the association between the kindergarten teachers’ second turnover and genders was significant (*p <* 0.05). The association between the kindergarten teachers’ second turnover and marital status was significant (*p <* 0.05). The association between the kindergarten teachers’ second turnover and kindergarten type was significant (*p <* 0.05). The association between the kindergarten teachers’ second turnover and educational background was significant (*p <* 0.05).

The kindergarten teachers that resigned thrice are 8.41%. The third turnover of kindergarten teachers occurred after 8.74 years of teaching experience. As shown in Table 3, the association between the kindergarten teachers’ third turnover and genders was significant (*p <* 0.05). The association between the kindergarten teachers’ third turnover and marital status was significant (*p <* 0.05). The association between the kindergarten teachers’ third turnover and kindergarten type was insignificant (*p >* 0.05). The association between the kindergarten teachers’ third turnover and educational background was significant (*p <* 0.05).

### 3.2 Influencing factors of kindergarten teachers’ different frequency turnover

Table 5 shows the Chi-square test results of the different frequency turnover of kindergarten teachers associated with eight influencing factors. Occupational stress (*p <* 0.01), the social context of kindergarten (*p <* 0.01), occupational ambition (*p <* 0.01), emotional attachment (*p <* 0.01), management mechanism (*p <* 0.01), reward(*p <* 0.05), organizational support (*p <* 0.01), and self-efficacy(*p <* 0.01) were significantly correlated with kindergarten teachers’ first, second, and third turnover.

**Table 5:** The different frequency turnover of kindergarten teachers association with eight influencing factors.

	First turnover			Second turnover			Third turnover		
	*χ* ^2^	p	Phi	*χ* ^2^	p	Phi	*χ* ^2^	p	Phi
Occupational stress	229.97	0.00	0.23	274.35	0.00	0.54	104.77	0.00	0.43
Social context of kindergarten	307.78	0.00	0.53	160.94	0.00	0.31	86.41	0.00	0.38
Occupational ambition	258.40	0.00	0.39	133.44	0.00	0.22	30.68	0.00	0.12
Emotional attachment	273.27	0.00	0.48	66.53	0.00	0.09	36.45	0.00	0.17
Organizational support	32.03	0.00	0.06	101.14	0.00	0.20	14.28	0.00	0.03
Management mechanism	259.23	0.00	0.42	262.83	0.00	0.48	333.67	0.00	0.68
Reward	568.25	0.00	0.65	187.89	0.00	0.41	55.57	0.00	0.26
Self-efficacy	259.07	0.00	0.41	144.03	0.00	0.28	200.11	0.00	0.57

The multivariate analysis via binary logistic regression found that occupational stress (*p <* 0.01), the social context of kindergarten (*p <* 0.05), occupational ambition (*p <* 0.01), emotional attachment (*p <* 0.01), management mechanism (*p <* 0.05), reward(*p <* 0.05), and self-efficacy(*p <* 0.01) were significantly related to kindergarten teachers’ first turnover. However, organizational support (*p >* 0.05) did not affect kindergarten teachers’ first turnover (Table 6).

**Table 6:** The binary logistic regression of influencing factors of kindergarten teacher turnover.

	First turnover-non turnover			Second turnover-non turnover			Third turnover-non turnover		
	B	OR	OR (95% CI)	B	OR	OR (95% CI)	B	OR	OR (95% CI)
Occupational stress	0.85**	2.34	1.89–2.92	1.30**	3.65	2.94--4.54	0.87**	2.39	1.91–2.99
Social context of kindergarten	−0.62**	0.54	0.43–0.68	−0.21*	1.24	1.01–1.51	−0.15	0.86	0.67–1.09
Occupational ambition	0.50**	1.65	1.36–2.01	0.41**	1.51	1.21–1.87	0.45**	1.58	1.23–1.99
Emotional attachment	1.06**	2.71	2.31–3.62	0.06	0.94	0.74–1.19	0.21	0.82	0.62–1.08
Organizational support	−0.01	0.97	0.77–1.31	−0.57**	1.76	1.29–2.40	−0.33*	1.39	1.05–1.83
Management mechanism	−0.23*	1.26	1.01–1.58	−0.37**	1.44	1.12–1.85	−0.68**	1.99	1.57–2.52
Reward	−1.76**	5.79	4.49–7.47	−0.86**	2.35	1.92–2.90	−0.75**	2.11	1.67–2.66
Self-efficacy	−0.45**	0.64	0.49–0.83	0.13	1.14	0.91–1.44	−0.05	1.05	0.82–1.34
Kindergarten types (Hu and Cai, 2022)	2.41**	4.06	1.96–8.40	0.90**	0.41	0.23–0.71	0.86*	2.35	1.29–4.29
Genders (Hu and Cai, 2022)	−1.14*	0.32	0.10–1.00	−2.75**	5.66	4.51–20.98	−2.40**	0.09	0.03–0.29
Educational backgrounds (Hu and Cai, 2022)	−0.54*	0.58	0.33–1.00	−0.35	1.42	0.78–2.60	−0.46	0.63	0.31–1.30
Marital status (Hu and Cai, 2022)	−0.25	0.78	0.45–1.31	−0.71*	0.49	0.28–0.87	−1.59**	0.20	0.10–0.40
Hosmer-Lemeshow test	*χ*^2^ = 6.61, *p* = 0.58			*χ*^2^ = 7.33, *p* = 0.50			*χ*^2^ = 9.12, *p* = 0.33		
Nagelkerke *R*^2^	0.83			0.73			0.33		

*p < 0.05, **p < 0.01.

As shown in Table 6, occupational stress (*p <* 0.01), the social context of kindergarten (*p <* 0.05), occupational ambition (*p <* 0.01), organizational support (*p <* 0.01), management mechanism (*p <* 0.01), and reward(*p <* 0.01) were significantly related to kindergarten teachers’ second turnover. However, self-efficacy(*p >* 0.05) and emotional attachment (*p >* 0.05) did not affect kindergarten teachers’ second turnover (Table 5).

As shown in Table 6, occupational stress (*p <* 0.01), occupational ambition (*p <* 0.05), organizational support (*p <* 0.01), management mechanism (*p <* 0.05), and reward (*p <* 0.05) were significantly related to kindergarten teachers’ third turnover. However, self-efficacy (*p >* 0.05), the social context of kindergarten (*p >* 0.05), and emotional attachment (*p >* 0.05) did not affect kindergarten teachers’ third turnover (Table 5).

## 4 Discussion

### 4.1 The relationship between socio-demographic variables and kindergarten teacher turnover

This research showed that kindergarten teachers resigned once, twice, and thrice 24.59, 10.64, and 8.41%, respectively. These results are consistent with previous studies showing that kindergarten teachers had high turnover rates (Darling-Hammond, 2003; Van Geffen and Poell, 2014; Cabezas et al., 2017; Doromal et al., 2022). With the emphasis on early education, there is a need for more kindergarten teachers in many countries and regions (Hu and Cai, 2022). Compared with previous cross-sectional studies, this research presented kindergarten teachers’ first, second, and third turnovers. This result helped us to understand the changes in the continuous turnover of kindergarten teachers.

The results showed that each turnover of kindergarten teachers was significantly correlated with gender, consistent with hypothesis 1. Previous studies indicated male teacher turnover was higher than female teachers (Struyven and Vanthournout, 2014; Husain et al., 2016). In contrast, the specific impact of gender on the kindergarten teachers’ first, second, and third turnover remains unclear. The results illustrate the particular characteristics of Chinese kindergarten teacher turnover. The traditional division of labor in Chinese families is that men work more outside the home, and women care for the family. These require relative job stability for women to take on more domestic work, while the need for job stability for men is relatively weak (Hall et al., 2013). Moreover, male kindergarten teachers are in short supply in China, which leads to more excellent career choices for male kindergarten teachers. These may lead to genders being significantly correlated with kindergarten teachers’ first, second, and third turnover.

We found that marital status was significantly correlated with kindergarten teacher turnover, consistent with hypothesis 1. In detail, marital status was significantly correlated with kindergarten teacher turnover (Wang, 2022). Marital status reflects the responsibility of employees to their families. Married employees usually choose a more stable lifestyle. Even if the work is not satisfactory, they will not easily quit. Also, unmarried employees are likelier to yearn for freedom, and their turnover costs are lower. Therefore, marital status was significantly associated with kindergarten teachers’ first, second, and third turnover.

The results demonstrated that the kindergarten type was significantly correlated with kindergarten teachers’ first and second turnover. Hypothesis 1 could be partially rejected. Some studies found that teacher turnover in private schools was significantly higher than in public schools (Li and Zheng, 2021; Siddiqui and Shaukat, 2021). Public kindergartens’ salaries, support, and environment are generally higher than private kindergartens (Tian, 2019). Chinese kindergarten teachers are more inclined to work in public kindergartens to pursue a better occupational life. Kindergarten teachers may be more concerned about the kindergarten type in the first and second turnover. The first and second turnovers may satisfy the occupational needs of kindergarten teachers who care about the kindergarten type. Therefore, these kindergarten teachers’ third turnover was unrelated to the kindergarten type.

This research found that the educational background was significantly correlated with kindergarten teachers’ second and third turnover. Hypothesis 1 could be partially rejected. According to human capital theory, kindergarten teachers’ educational experience positively correlates with job opportunities (Kantarci and Van Soest, 2008). Generally, kindergarten teachers in the first turnover needed higher educational experience. All the kindergarten teachers with varying educational backgrounds had low career opportunities in the first turnover. Subsequently, kindergarten teachers accumulated more work experience in the second and third turnover. Therefore, kindergartens were more likely to choose highly educated kindergarten teachers with the same work experience.

### 4.2 The influence of eight factors on kindergarten teacher turnover

The results showed that occupational stress, reward, management mechanisms, and ambition consistently affected kindergarten teachers’ first, second, and third turnover. Hypothesis 2 could be partially rejected. The findings were similar to some studies. For instance, new teachers refused to continue teaching due to needing more support, high occupational stress, and intense competition within schools (Struyven et al., 2012). Job satisfaction, school policies, family attachment, occupational stress, and prospects were reasons for new teachers to exit (Hancock and Scherff, 2010; Struyven and Vanthournout, 2014). Moreover, self-efficacy, the social context of the school, salary, and occupational stress significantly affected teachers’ retention and turnover intention (Hughes, 2012; McConnell, 2017). Compared with previous studies, This research explored the influence of multiple factors on kindergarten teachers’ first, second, and third turnover. Overall, the factors affecting each turnover of kindergarten teachers have some common characteristics and some different characteristics.

Occupational stress and reward are crucial for teacher turnover (Hughes, 2012; Reininger, 2012; Struyven et al., 2012; McConnell, 2017). This research analyzed the influencing factors of different turnover frequencies and found that these factors continue to affect the subsequent turnover of kindergarten teachers. Rational choice theory indicates that individuals use their strengths and value to measure the gains and losses of exchange behavior, thus seeking to maximize their utility (Coleman, 1994). Turnover is a rational choice for kindergarten teachers. Occupational stress and reward affect the living quality of kindergarten teachers. Kindergarten teachers would expect a balance between occupational reward and occupational stress by rational choice. Most kindergarten teachers’ turnover happens after this balance is broken. For example, a survey showed that 44.47% of kindergarten teachers in China had high work stress and common rewards (Gong et al., 2020). This unbalanced strategy leads to the turnover of kindergarten teachers. Increasing labor performance may be an effective way to reduce teacher turnover.

We found that occupational ambition continually affects the turnover of kindergarten teachers, which is different from a previous study (Hall et al., 2013). Human resource management theory indicates that most knowledgeable employees have career aspirations (Guest, 2001). Kindergarten teachers have high occupational aspirations and desire high occupational achievements. These occupational aspirations depend on career experience, professional understanding, individual ability, talents, and value orientation. Occupational aspirations may be changed with the objective environments and individual awareness (Guest, 2001). Furthermore, work experience can improve kindergarten teachers’ teaching ability and understanding of their occupation, thus increasing their occupational aspirations. Therefore, kindergarten teachers may look for suitable kindergartens to satisfy their occupational aspirations, which may result in the turnover of kindergarten teachers.

This research found that management mechanisms continued to affect the turnover of kindergarten teachers. School management is a basic tool to ensure teachers’ work behavior, work efficiency, and individual rights (Quartz et al., 2005; Du, 2017). Kindergarten teachers will choose to resign if the management mechanisms of kindergarten obstruct their work or are difficult to follow.

The social context of kindergarten was significantly related to kindergarten teachers’ first and second turnover. Some studies found that the social context of kindergarten affected kindergarten teacher turnover (Hughes, 2012; McConnell, 2017). The high social reputation of the schools brings honor to teachers (Hughes, 2012). Kindergarten teachers prefer to work in schools with high social reputations, especially for teachers with high professional abilities. In the first and second turnover, kindergarten teachers will cautiously clarify the kindergartens’ social context to avoid the third turnover. Moreover, a study showed that organizational support affects teacher turnover (Fulbeck, 2014). This research found organizational support was significantly related to kindergarten teachers’ second and third turnover but did not affect the first turnover. Perceived organizational support can evaluate the organization’s care for teacher contribution, value, and happiness (Lin et al., 2006). When the first turnover happened, the average teaching experience of kindergarten teachers was only 2.03 years. Teachers may need to care more about the recognition from kindergarten due to the short teaching time. When the teaching experience gradually improves, kindergarten teachers are more likely to demand the kindergarten’s recognition. Accordingly, kindergartens should pay more recognition and support to teachers with more teaching experience to prevent second and third turnover.

Previous studies found that emotional attachment and self-efficacy affected teacher turnover (Cassettari et al., 2014; Tiplic et al., 2015). In this research, emotional attachment and self-efficacy were significantly related to kindergarten teachers’ first turnover but not the second and third turnover. In detail, self-efficacy is the speculation and judgment of individuals, which is crucial to evaluating the efficiency of individuals completing certain behaviors (Lian, 2007). For the first job, kindergarten teachers may reduce their psychological capital when the teachers lack confidence in completing the kindergarten work (Ladd, 2011). Subsequently, the kindergarten teachers may leave and find another kindergarten that matches their self-assessment. Additionally, high emotional attachment may promote individuals’ demand for family emotional support, and individuals find it difficult to break away from the family (Molly and David, 2016). The emotional support from family may lead to some kindergarten teachers choosing a job near the family. These teachers may flow slowly to avoid losing family emotional support.

### 4.3 Limitations and future directions

This research emphasized the current situation and influencing factors of kindergarten teachers’ first, second, and third turnover. This research used a simple random sampling method, and 1,118 Chinese kindergarten teachers voluntarily participated in this survey. This is a convenient sample. Therefore, this survey is difficult to avoid selection effects. Some scientific and effective sampling methods may be found in the future, and more teachers may be invited to complete this survey. Secondly, this survey collected all information in a self-reported manner. The known quality of self-reporting limits this method. In particular, kindergarten teachers need to recall the kindergarten information they worked on in one frequency survey. Therefore, multiple-frequency surveys should be considered in the future. The questionnaire used in This research passed exploratory and confirmatory factor analysis. Some specific measurement tools and methods for kindergarten teacher turnover should be developed. Thirdly, This research analyzed the effects of 8 factors on kindergarten teacher turnover. In the future, it is necessary to explore the key factors that affect kindergarten teacher turnover, especially for the organization and management of kindergartens. Furthermore, future research should control the interfering factors in the survey. Lastly, This research was cross-sectional and lacked a longitudinal survey in the research design. Therefore, longitudinal surveys should be added in subsequent studies to improve the reliability of conclusions.

## 5 Conclusion

This research found that kindergarten teachers’ first, second, and third turnovers were frequent. The continuous high turnover of kindergarten teachers needs to be paid more attention to. This research found that socio-demographic variables were correlated with kindergarten teacher turnover. Kindergartens are more likely to recruit women or married people. Kindergartens should pay more attention to private and highly educated teachers to reduce turnover. This research found the persistent factors that affect kindergarten teacher turnover. Kindergartens must reduce teachers’ pressure, improve rewards and management mechanisms, and allow teachers to display their talents. In summary, this research provides empirical support for effective strategies to reduce the frequent turnover of kindergarten teachers.

## Data availability statement

The raw data supporting the conclusions of this article will be made available by the authors, without undue reservation.

## Ethics statement

The studies involving humans were approved by the Ethics Committee of the School of Education, Beihua University. The studies were conducted in accordance with the local legislation and institutional requirements. The participants provided their written informed consent to participate in this study. Written informed consent was obtained from the individual(s) for the publication of any potentially identifiable images or data included in this article.

## Author contributions

XR: Funding acquisition, Visualization, Writing – original draft, Writing – review and editing. ZY: Conceptualization, Methodology, Resources, Supervision, Writing – original draft. ZZ: Conceptualization, Methodology, Resources, Supervision, Writing – original draft. JC: Data curation, Investigation, Software, Validation, Writing – original draft. YT: Data curation, Investigation, Software, Validation, Writing – original draft.
